# Immediate and Short-Term Outcome of Percutaneous Atrial Septal Defects Closure in Adult Patients

**DOI:** 10.7759/cureus.11165

**Published:** 2020-10-26

**Authors:** Muhammad A Khan, Hussain Korejo, Arshad Sohail, Abdul S Shaikh, Najma Patel

**Affiliations:** 1 Paediatric Cardiology, National Institute of Cardiovascular Diseases, Karachi, PAK; 2 Paediatric Cardiology, Rehman Medical Institute, Peshawar, PAK

**Keywords:** atrial septal defect secundum, percutaneous atrial septal defects closure, congenital heart defects, adult

## Abstract

Background

Atrial septal defect secundum (ASD II) is the commonest of the congenital heart diseases in the adult population and the closure of ASD II causes a significant improvement in hemodynamics and the clinical status of the patient. However, it carries certain risks, especially the development of post-closure pulmonary edema and congestive cardiac failure, which may lead to death. Therefore, this study was designed to share our experience and to evaluate the immediate and short-term outcomes of percutaneous closure of ASD II in adult patients presenting at a tertiary care cardiac center in Karachi, Pakistan.

Methodology

In this study, we included 30 adult (≥ 40 years) patients who underwent percutaneous ASD II closure at the pediatric cardiology department of the National Institute of Cardiovascular Diseases (NICVD), Karachi, Pakistan, between June 1, 2017, and July 31, 2019. Data for this study were extracted from a prospectively collected departmental database. Extracted data for this study consisted of demographic profile, comorbid conditions, echocardiographic findings, cardiac catheterization data, pre and post six-month electrocardiographic findings, and New York Heart Association (NYHA) Functional Classification (FC).

Results

Out of 30 adult patients, 18 (60%) were female. The mean age of the patients at the time of ASD closure was 51.43 ± 7.09 years, ranging between 40 and 67 years. None of the patients had any post-intervention complications. Mean systolic pulmonary artery pressure (SPAP) on cardiac catheterization pre-implantation was 49.8 ± 16.3 mmHg range was 20-90 mmHg while SPAP immediately after device implantation was 37 ± 11.4 range 20 to 65 mmHg with p<0.001. Mean SPAP on pre-catheterization echocardiography was 58.8 ± 14.3, range 30-95 mmHg, while after six months, the mean SPAP was 34.5 ± 7.2, range 20-45 mmHg, with p<0.001 in 28 (93.3%) patients (in two patients, there was no TR). There was no mortality up till six months after the intervention. The functional class (FC) after six months of the procedure improved in most, 90% (27), of the patients.

Conclusion

There were no complications immediately after the procedure. After six months, post-procedure SPAP decreased to < 50 mmHg in the majority of patients (96.6%). Functional class (FC) improved in most (90%) of the adult (≥ 40 years) patients undergone percutaneous atrial septal defects closure. Therefore, percutaneous closure of ASD II is a safe and effective procedure for adult patients.

## Introduction

Atrial septal defects (ASD II) account for approximately 10% of all congenital heart defects (CHD) [1-5]. However, in the adult population, it accounts for 30%-40% of CHD. Patients with ASD II remain asymptomatic until late, when they present in adult life with palpitation, decreased exercise tolerance, and arrhythmia [3-6]. Therefore, the closure of ASD with a significant left to right shunt results in improved functional capacity and improves arrhythmias, which becomes more responsive to treatment [3,5,7]. However ASD closure in adult patients has certain risks, specifically may result in pulmonary edema and heart failure and may lead to death. This is due to an abrupt elevation in left ventricular preload following ASD closure in non-compliant LV, which may be secondary to mechanical response to chronic right ventricle (RV) volume overload, which results in compression of the left ventricle (LV), abnormal diastolic-systolic relations of the interventricular septum, and chronic LV underfilling with increased left atrial (LA) pressure, resulting in so-called "masked LV restriction,″ or may be secondary to comorbid diseases such as systemic hypertension, coronary heart disease, or age-related LV diastolic dysfunction. Therefore, cardiac catheterization before closing ASD is necessary and if left ventricular end-diastolic pressure (LVEDP) is found high, then the balloon occlusion test should be done before closure to avoid this complication [8]. Different protocols for the detection and management of high-risk patients are used [9], however, we used to start anti-failure treatment, including angiotensin-converting enzyme (ACE) inhibitors and diuretics, when the patient was referred to us to avoid doing cardiac catheterization twice so the radiation exposure is reduced. Transcatheter closure of isolated secundum ASD is an established mode of treatment in adult patients [3,10-11]. This study was designed to evaluate the immediate and short-term outcome of percutaneous ASD II closure in adult patients presenting at a tertiary care cardiac center in Karachi, Pakistan.

## Materials and methods

Between June 1, 2017, and July 31, 2019, a total of 150 procedures of ASD II closure by device were performed at the pediatric cardiology department of the National Institute of Cardiovascular Diseases (NICVD) Karachi, Pakistan. Out of these 150 patients, 30 patients were 40 or above 40 years of age. After departmental and ethical review committee approval (ERC-41/2019), data for this study were extracted from a prospectively collected departmental database of patients with ASD II who underwent device closure. In accordance with the Declaration of Helsinki, written informed consent was obtained as part of our institutional filing system, and consent regarding the use of collected information for research purposes was obtained from all the patients. Patient records indicating refusal of consent were excluded from this study.

Inclusion criteria for the study were either gender, age 40 years or above, diagnosed with isolated secundum ASDs, given consent, underwent percutaneous ASD closure, and completed the six-months post-procedure functional outcome questionnaire. All patients with ASD II were diagnosed and managed as per the guidelines and institutional set protocols. All the procedures were performed by a consultant cardiologist with more than five years of working experience. Extracted data for this study consisted of age, gender, pre-procedural presenting symptoms (shortness of breath, fatigue, and palpitations, etc.), comorbid conditions (hypertension, diabetes mellitus, or myxedema), echocardiographic findings of RV volume overload, including RV size and paradoxical septal motion and function, LV systolic and diastolic function, ASD size, numbers, and rims, and any associated lesion and assessment of pulmonary arterial hypertension (PAH) and mitral regurgitation (MR) were recorded. Catheterization parameters, including LA mean pressure (mmHg), left ventricular end-diastolic pressure (LVEDP), pulmonary artery pressure systolic (SPAP), diastolic and mean (mmHg), and pulmonary vascular resistance (PVR) in Woods unit. After device closure, echocardiography and electrocardiograms (ECGs), as well as clinical assessments, were done after 24 hours, then at one month, three months, six months, and one year and then yearly. All patients were prescribed dual antiplatelet therapy (aspirin and clopidogrel) and anti-failure therapy. However, for this study, we include assessment after six-month intervention outcomes only. Six months post-intervention echocardiographic findings, including RV size and function, LV systolic and diastolic function, assessment of PAH, residual shunt, any mitral regurgitation, and device position were recorded. Electrocardiographic findings (sinus rhythm, persistent, or intermittent atrial fibrillation, etc.) and NYHA functional classification (FC) were recorded.

All patients had pre-procedure transesophageal echocardiography (TEE) for assessment of suitability for the device. Patients with all rims ≥5 mm except the aortic rim were selected. All procedures of ASD II closure by device were performed under general anesthesia. Cardiac catheterization data were collected, and a coronary angiogram was done in patients older than 50 years. None had any coronary artery diseases. TEE was performed before and during the procedure. ASD sizing was done on TEE and in three patients with floppy rims, sizing done with compliant sizing balloon with the stop-flow technique. Balloon occlusion test was performed in 16 (53.33%) patients with LVEDP >15 mmHg. In all five (16.6%) patients with pre- or post-occlusion LVEDP >20 mmHg, fenestration in the ASD device was made. ASD device size was calculated by adding 4 mm to the maximum diameter taken. An intra-procedural intravenous (IV) weight-adjusted dose of heparin was given. A multipurpose A 2 (MPA 2) 6 French catheter was advanced through the femoral vein into the right upper pulmonary vein (RUPV), then a J-tipped super-stiff guide wire 0.035 was advanced and placed in the RUPV.

A 9 to 12 French delivery system was used to deliver the device. The device was advanced into the RUPV, the LA disc was partially deployed, the right atrial (RA) disc deployed in the right atrium, and then the device pulled. In three patients, the balloon assisting technique was used, and in three patients, the LA disc was deployed in the LA roof. Two patients with multiple ASDs had closure with a single device. LVEDP was measured after device closure via a pigtail catheter advanced retrogradely via the femoral artery. A Minnesota wiggle was done after deploying both discs while the device was still attached to the delivery cable. After confirming the proper position on TEE, the device was released.

Collected data were analyzed using IBM SPSS Statistics for Windows, Version 21.0. (IBM Corp., Armonk, NY). Mean ± standard deviations (SD) were calculated for the continuous variables, and frequency and percentages were calculated for categorical variables.

## Results

Out of the 30 adult patients who underwent percutaneous atrial septal defects closure, 60% (18) were female. The mean age of the patients at the time of ASD closure was 51.43 ± 7.09 years ranged 40 to 67 years of age. The most common symptoms at presentation were shortness of breath in 90% (27) and fatigue in 73.3% (22) of the patients. The majority of the patients, 73.3% (22), were in NYHA functional class II and 20% (six) patients were in functional class III. Among other comorbid conditions, 33.3% (10) were hypertensive and 6.6% (two) were diabetic. Two patients (6.6%) had persistent atrial fibrillation and two patients (6.6%) had intermittent atrial fibrillation at presentation. Detailed baseline and demographic characteristics are presented in Table 1.

**Table 1:** Baseline and demographic characteristics of adult patients who have undergone percutaneous atrial septal defects closure

Demographic feature	N = 30
Gender	
Male	40% (12)
Female	60% (18)
Age (years)	
Mean ± standard deviation (years)	51.43 ± 7.09
Range (years)	40 to 67
40 to 50 years	60% (18)
51 to 60 years	33.3% (10)
61 to 67 years	6.6% (2)
Symptoms at presentation	
Shortness of breath	90% (27)
Fatigue	73.3% (22)
Palpitations	46.6% (14)
Limited exercise capacity	30% (9)
New York Heart Association (NYHA) Functional Class	
I	6.6% (2)
II	73.3% (22)
III	20% (6)
Co-morbid conditions	
Hypertension	33.3% (10)
Hypertension + Diabetes Mellitus	6.6% (2)
Diabetes Mellitus + Hypertension + Myxedema	3.3% (1)
Cerebrovascular accident (CVA)	3.3% (1)
Transient ischemic attack (TIA)	3.3% (1)
Electrocardiography	
Sinus rhythm	86.7% (26)
Persistent atrial fibrillation	6.6% (2)
Intermittent atrial fibrillation	6.6% (2)

Mean SPAP on cardiac catheterization pre-implantation was 49.8 mmHg ± 16.3, range 20-90 mmHg, while SPAP immediately after device implantation was 37 ± 11.4, range 20 to 65 mmHg, with p-value <0.001.

The pulmonary blood flow (Qp) to systemic blood flow (Qs) ratio (Qp:Qs) was ≥ 3:1 in all the patients. Mean PVR was 4.37 ± 1.22; PVR was <3 Woods unit in 23 patients (76%), 3-6 Woods unit in six (17.1%), and more than 6 Woods units in one (3.3%). This patient had a PVR of 10 Woods units at room air, which decreased after a hyperoxia test to 6 Woods units. Hemodynamics data obtained pre- and post-ASD closure are presented in Table 2.

**Table 2:** Hemodynamics during cardiac catheterization pre and post ASD device implantation LVEDP: left ventricular end-diastolic pressure; ASD: atrial septal defects

Parameters	N = 30	N=30
LVEDP		
Mean ± standard deviation (mmHg)	16.5 ± 4.73	17.4±4.67
Range (mmHg)	8 to 26	8 to 26
≤ 10 mmHg	5 (16.6%)	04 (13.3% )
11-15 mmHg	09 (30%)	08 (26.6% )
16-20 mmHg	13 (43.3%)	13(43.3%)
21-26 mmHg	03 (10%)	05(16.6% )
Systolic pulmonary artery pressure		
Mean ± standard deviation (mmHg)	49.8± 16.3	37 ± 11.42
Range (mmHg)	20-90	20-65
≤25 mmHg	03 (10%)	08 (26.6% )
26 -60 mmHg	20 (66.6% )	21 (70%)
> 60 mmHg	07 (23.3%)	01 (3.3% )

The balloon occlusion test was performed in 16 (53.33%) patients with LVEDP >15 mmHg. There was an increase in LVEDP in three patients; in two patients with pre-occlusion LVEDP of 18 and 16 mmHg, it increased to 22 mmHg in both, and in one patient with LVEDP of 22 mmHg, it increased to 26 mmHg. In all five (16.6%) patients with pre- or post-occlusion LVEDP >20 mmHg, fenestration in the ASD device was made.

**Figure 1:**
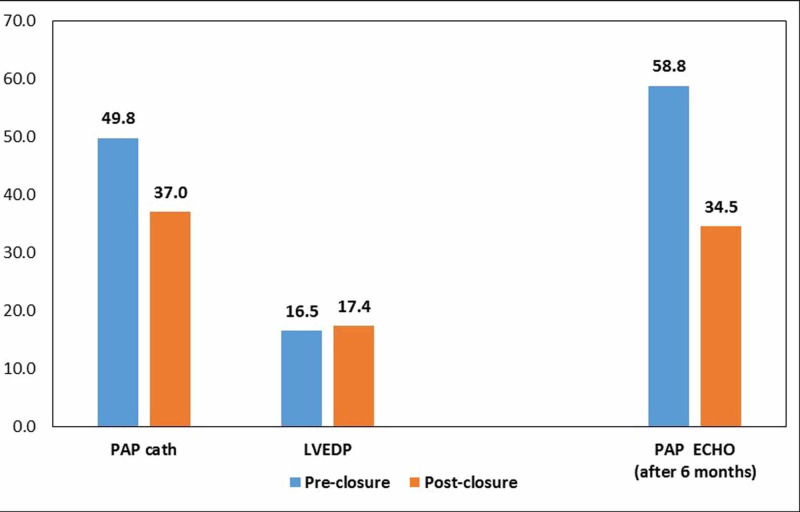
Pre- and post-percutaneous atrial septal defects (ASD) closure left ventricular end-diastolic pressure (LVEDP) and pulmonary artery pressure (PAP) on cardiac catheterization and echocardiography

ASD size on TEE was 18 to 34 mm, with a mean ± SD of 27.71 ± 4.25. Two patients had multiple ASDs. Device size was 22 to 38 mm, with a mean ± SD of 31.41 ± 3.94 mm, and ASD closure was successful in all patients. None of the patients had any post-intervention complications. Mean LVEDP pre-occlusion was 16.5 ± 4.7 mmHg (8-26 mmHg), which increased to 17.43 ± 4.67 mmHg, with a p-value of <0.001. Four patients showed an increase in LVEDP after device closure, who had pre-closure LVEDP of <20 mmHg. The pre-closure LVEDP of these patients was 8, 12, 16, and 18 mmHg, which increased to 14, 18, 22, and 24 mmHg, respectively. However, none of them showed any clinical manifestation. Patients who had an initial pre-closure LVEDP of >20 mmHg did not have an increase in LVEDP after device closure, which may be due to fenestration in the device. 25 (83.3%) were extubated immediately after the procedure. All five patients with LVEDP of >20 mmHg pre or post-closure were kept electively on a ventilator for a few hours. Two patients, with intermittent atrial fibrillation (AF) after positioning the device had cardioversion while the cable was still attached and converted to sinus rhythm and did not develop AF later, were kept on medication for a few months, and two patients with persistent atrial fibrillation had rate control after the intervention. Mean SPAP on echo pre-catheterization was 58.8±14.3 mmHg, range 30-95 mmHg, while after six months, the mean SPAP was 34.5 ± 7.2 mmHg, range 20-45 mmHg, with a p-value of <0.001 in 28 (93.3%) patients (in two patients, there was no TR). SPAP decreased to <50 mmHg in 29 (96.6%) patients. Figure 1 represents the pre and post-ASD device closure LVEDPs and pulmonary artery pressures on cardiac catheterization and echocardiography.

**Figure 2:**
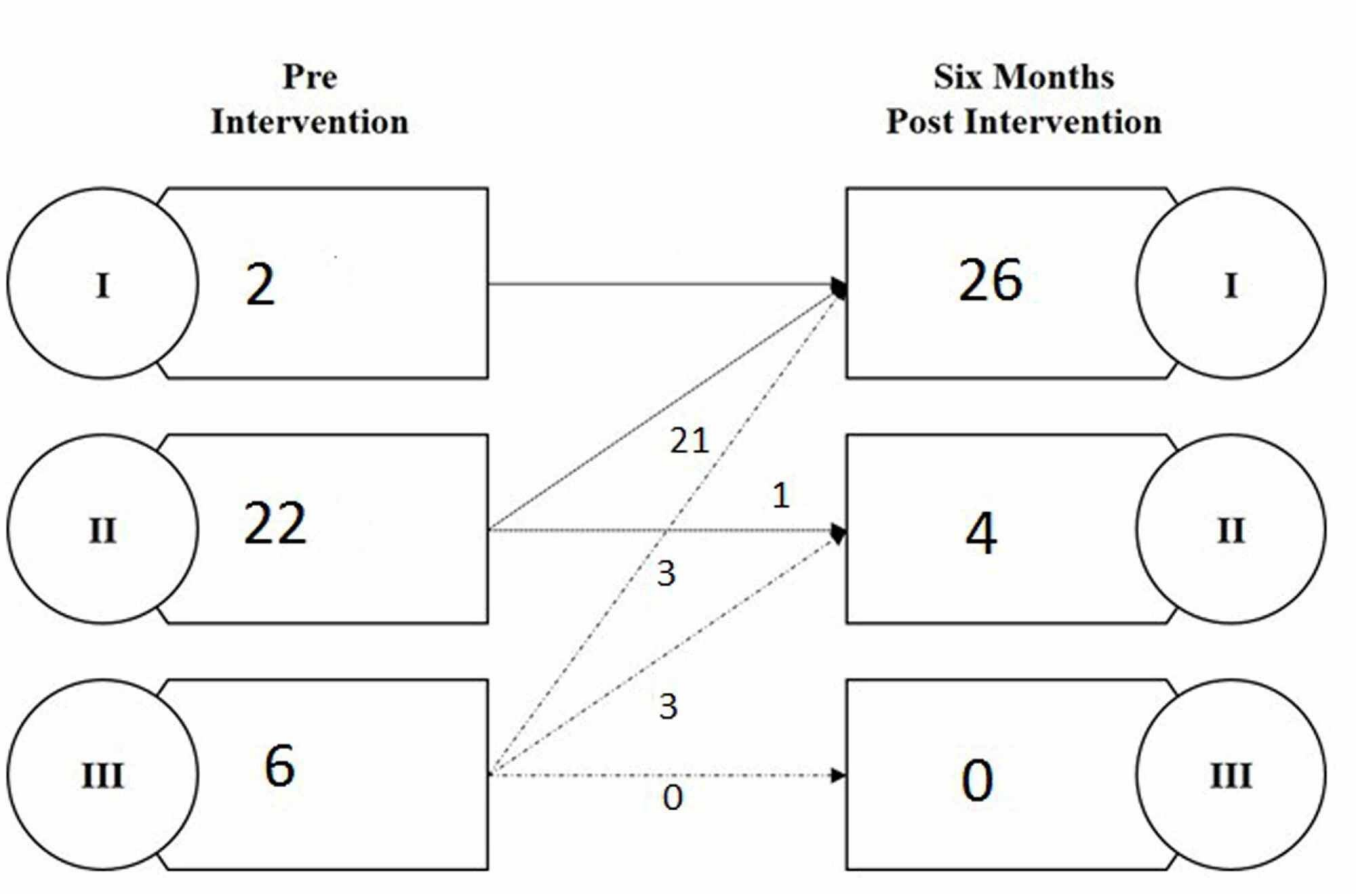
Pre and six months post-intervention New York Heart Association (NYHA) functional classification of adult patients who have undergone percutaneous atrial septal defects closure

There were no mortalities up till six months after the intervention. The functional class (FC) after six months of the procedure improved in most of the patients, 90% (27), and remains the same in 10% (3) patients. Pre and six months post-intervention NYHA functional classifications are presented in Figure 2.

## Discussion

ASD is one of the common congenital heart diseases in the adult population and if left untreated, can cause right-sided heart failure, left ventricular failure, atrial fibrillation or flutter, and irreversible severe pulmonary artery hypertension [3,6-7,11]. This study was conducted to evaluate the immediate and short-term outcomes of percutaneous atrial septal defects closure in adult patients. The main findings of the study were that there were no adverse outcomes of percutaneous ASD closure, no post-procedure new-onset arrhythmia, pre-procedure intermittent atrial fibrillation converted to sinus rhythm, and persistent atrial fibrillation had rate control after the intervention. Functional class improved after six months of the procedure in the majority of the patients.

Supporting the finding of our study, various past studies have reported a significant improvement in the functional class of the adult (≥40 years) patients after percutaneous atrial septal defects (ASD) closure in adult patients [3,6-7,12-13]. It is also reported that ASD device closure leads to favorable and positive cardiac remodeling [6-7,12] and showed systematic regression and improvement of RV enlargement, SPAP, and RV and LV function [3,7,13-14].

Percutaneous ASD closure in adults (≥40 years) was found to be safe, cost-effective, and efficient [15-16]. A recent study by Alnasser et al. reported that the mortality rate after ASD device closure was not greater than that of individuals from the age- and sex-matched general population, and the mortality rate after a median of nine years was significantly not different between surgical and percutaneous ASD closure [17]. Similarly, various other studies have established the effectiveness of device closure in adults. A population-based, retrospective cohort study conducted by Kotowycz et al. compared the long-term safety and effectiveness of surgical and transcatheter ASD closure and concluded that percutaneous ASD closure is not inferior to the surgical closure of ASD in adult patients [18]. A study conducted by Khan et al. in the local population concluded that ASD device closure is safe and convenient [19]. Along with the added advantage of the less invasive nature of device closure, it was found to be cost-effective and associated with shorter hospital stay and less number of post-procedure complications [20-24].

In our study, all the patients were free of any post-procedure in-hospital major complications, similarly, either low or no complications were reported by various studies from different parts of the world [3,6-7,12-13,15]. In our study, mean PAP decreased to <50 mmHg in 96.6% of the patients, and functional class improved in 90% of the patients, which is higher than the 74% reported by Akagi et al. [7].

ASD device closure in adults is a safe, effective, and less invasive alternative, and with proper and careful prior hemodynamic assessment of patients, it can produce good outcomes. Some contraindications for the procedures are severe irreversible pulmonary arterial hypertension (PAH), severe left ventricular dysfunction, pregnancy, small ASDs ˂5 mm without any evidence of right ventricular volume overload (RVVO), and Qp:Qs ratio ≤0.7. A single center-based study, with small sample size, is the key limitation of this study.

## Conclusions

The functional class after six months of the procedure improved in most (90%) of the adult (≥40 years) patients who underwent percutaneous atrial septal defects closure, post-procedure mean pulmonary artery pressure decreased to <50 mmHg in the majority (96.6%) of patients, there was no new-onset arrhythmia and pre-procedure intermittent atrial fibrillation converted to sinus rhythm, and persistent atrial fibrillation had rate control after the intervention. Therefore, percutaneous atrial septal defects closure is a safe and effective procedure for adult patients.
